# Biofoam of Spittlebug, *Poophilus costalis* (Walker): Preferential Sites, Temperature Regulation, Chemical Composition and Antimicrobial Activity

**DOI:** 10.3390/insects12040340

**Published:** 2021-04-12

**Authors:** Kitherian Sahayaraj, Balakrishnan Saranya, Samy Sayed, Loko Yêyinou Laura Estelle, Koilraj Madasamy

**Affiliations:** 1 Crop Protection Research Centre, Department of Zoology, St. Xavier’s College, Palayamkottai, Tirunelveli 627002, Tamil Nadu, India; velsaran0156@gmail.com (B.S.); madasamy041992@gmail.com (K.M.); 2 Department of Science and Technology, University College-Ranyah, Taif University, B.O. Box 11099, Taif 21944, Saudi Arabia; samy_mahmoud@hotmail.com; 3 Laboratory of Applied Entomology, National High School of Biosciences and Applied Biotechnologies (ENSBBA), National University of Sciences, Technologies, Engineering and Mathematics, Dassa-Zoumé BP 14, Benin; lokoestelle@yahoo.fr

**Keywords:** spittlebug, biofoam, biochemistry, composition, host plants, antimicrobials

## Abstract

**Simple Summary:**

Spittlebugs produce foam in either nodes or internodes on stems of *Theporsia purpurae* (100%) and *Mimosa pudica* (100%), to establish and protect their young. However, this reduces the photosynthetic capacity of the plants. The surface area of the foam is higher for *Lawsonia inermis* (18.15 cm^2^) than other plants. The number of nymphs in each piece of foam varied from 1 to 3 in this study. The foam was cooler than the external environment for all the tested plants (*p* < 0.0001), except *T. purpurae* and *M. pudica* (*p* > 0.05). The biofoam consists of carbohydrates, amino acids, proteins and fatty acids. A saturated fatty acid, octadecanoic acid, was more abundant (88.33%) in the biofoam of *Poophilus costalis*. The biofoam showed strong antibacterial activity against *Staphylococcus aureus* than that of other species, similar to that of chloramphenicol against *Pseudomonas fluorescens*.

**Abstract:**

The foam produced by nymphs of *Poophilus costalis* on eleven different host plants belonging to eight families on St. Xavier’s College campus in India was studied over five months. The chemical composition and antimicrobial activity of these biofoams were investigated. The results revealed that *P. costalis* preferred *Theporsia purpurea* and *Mimosa pudica* for laying their eggs and producing foam, over the other tested plants. *P. costalis* produce their foam on either nodes or internodes on monocotyledons (30%) (*p* < 0.05), whereas on dicotyledons, they produce more foam on the stems (63.8%) than on the leaves (6.2%) (*p* < 0.01). The number of nymphs in each piece of foam from *P. costalis* varied from 1 to 3 (mean = 1.8 per plant). They produced their foam (5.7 to 45.2 cm) from the ground level on a plant. The length and breadth of a piece of foam ranged from 1.0 to 3.9 cm and 0.6 to 4.7 cm, respectively. The foam tended to be cooler than the environment. Qualitative profiling showed that the foam consists of carbohydrates, including maltose; trypsin; amino acids; protease. The foam was also analyzed using a spectrophotometer, Fourier transform infrared spectroscopy (FT-IR), gas chromatography–mass spectroscopy (GC-MS), and high-performance liquid chromatography (HPLC). The antimicrobial activity of the biofoam was the greatest against *Staphylococcus aureus*, the growth of which was reduced by 55.9 ± 3.9%, suggesting that the foam could be used as an antimicrobial product. However, no activities were observed against *Fusarium oxysporum* and *Candida albicans*.

## 1. Introduction

The spittlebug *Poophilus costalis* (Walker) (Hemiptera: Aphrophoridae) is an important crop pest widely distributed in African and Asian regions that feeds on more than 37 plant species [1]. This xylem-feeding pest has been observed to severely damage lavender [2], common sorrel [3] and medicinal and aromatic plants [4] in India, and sorghum in some West and Central African countries [5,6]. Ajayi and Oboite [5] noted that heavy infestation by *P. costalis* could kill seedlings and cause the stunting of mature plants. In addition, this spittlebug has the potential to act as a vector of plant diseases [7] and has been reported as a vector of rice yellow mottle disease [8]. Several methods including the use of synthetic chemical insecticides and biopesticides have been used to control this pest [6]. However, the foaming substance produced by the spittlebug nymphs, which protects them against predators and parasitoids and acts as thermal insulation [9], is an important obstacle in their management [10]. Indeed, the chemical composition of and beneficial organisms contained in the foam produced by spittlebugs protect the nymphs [11,12]. Moreover, [13] showed that the foam of spittlebugs has a thermoregulatory role, allowing these pests to resist extreme temperatures. Therefore, it would be interesting to evaluate the role of thermoregulation by the foam produced by *P. costalis*.

Very limited information is available about the chemical composition of the foam produced by spittlebug nymphs. A study [13] showed that palmitic acid, stearic acid, carbohydrates and protein were the main compounds of the foam produced by nymphs of the froghopper *Mahanarva fimbriolata* (Stål) (Hemiptera: Cercopidae). However, there is very little information on the biochemical composition of the foam of *P. costalis*; knowledge of such will permit the development of efficient strategies for fighting this pest. In addition, the identification and quantification of the chemical compounds of the foam produced by *P. costalis* nymphs will help to elucidateits diverse functions. In fact, some studies have shown that the foam produced by spittlebugs has diverse biological activities and can act as a repellent [11], antibiotic [12], antifungal and chitinase agent [14]. It would also be interesting to evaluate the antimicrobial activity of the foam of *P. costalis* nymphs to explore the possibility of using entomopathogenic organisms in the management of this pest.

Spittlebugs feed on various plants, and most them select specific plants for their development [15,16]. Although 37 potential host plants have been revealed for *P. costalis* [1], there is little information on the distribution and preferential exploitation of these host plants by its nymphal stages. This information is essential for the determination of the host plants allowing the development and abundance of *Philaenus spumarius* spittlebug [16]. Good knowledge about the plant selection of *P. costalis* is important for designing efficient strategies for managing this spittlebug. Therefore, this study aimed to record (1) the distribution of the foam of *P. costalis* in across various host plants (*Boehmeria cylindrical* L. (Urticaceae), *Cynodondactylon* L. (Poaceae), *Eragrostis amabilis* L. (Poaceae), *Ocimum americanum* L. (Lamiaceae), *Corchorus olitorius* L. (Malvaceae), *Lawsonia inermis* L. (Lythraceae), *Tridax procumbens* L. (Asteraceae), *Phyllanthus amarus* L. (Euphorbiaceae), *Clitoria ternatea* L. (Fabaceae), *Mimosa pudica* L. (Fabaceae), and *Tephrosia purpurea* L. (Fabaceae) present on St. Xavier’s College campus; (2) the occurrence and size of the foam produced by *P. costalis* in different organs of host plants; (3) the chemical composition of the foam produced by *P. costalis*; (4) the temperature between the environment and inner side of the foam produced by *P. costalis*; and (5) the antimicrobial activity of the foam produced by *P. costalis* on *Escherichia coli*, *Xanthomonas malvacearum*, *Xanthomonas citri*, *Staphylococcus aureus*, *Pseudomonas fluorescens*, *Fusarium oxysporum* and *Candida albicans*.

## 2. Materials and Methods

### 2.1. Field Work

The experiment was conducted inside St. Xavier’s College campus (Tamil Nadu, India) from November 2019 to March 2020. During the study period, the temperature ranged from 28 °C to 32 °C, and the humidity ranged between 71 and 84%. The light cycle was 13 h of light and 11 h of dark (L:D). The individual plants were monitored, and the populations of *P. costalis* were continuously recorded on 11 plant species bellowing to 8 families for at least three months. Uponeach observation, the following parameters were recorded: the height of the plant (cm) and the height (cm) at which the spittlebug produced its foam. The number of nymphs per spittle was assessed per individual host plant and per host-plant taxon, together with the position of the nymphs on the individual host plant (stem, node, and internode; leaf surface—upper or lower). The spittlebug samplings were conservative, and each instar was determined directly in the field based on the size and morphology of the nymphs. The length and breadth (cm) of the foam were recorded by using a 30 cm-length Camelin scale with a graduation of 10 mm. The amount of foam present in a plant and the spittlebug nymph stages dwelling in the foam were determined. The third, fourth and fifth nymphal instars were 2.5, 4.0 and 6.0 mm in size, respectively. The natural enemies (if any) of *P. costalis* nymphs under natural conditions were also recorded during the investigation period.

### 2.2. Ecophysiology of Foam and Spittlebug

Using a laboratory thermometer with a ±0.1 accuracy (Lab world, Hyderabad, India), the temperatures were recorded both inside and outside the foam found in the various plants. For recording the temperature inside the foam, the thermometer was slightly inserted into the foam, and after a minute, the temperature was noted. Any foam adhering to the thermometer was removed using paper before inserting it into another foam. The external temperature and humidity within a 5–7 cm radius of the foam were measured between 12 noon and1.00 p.m. Observations were made for 31 plants each for *T. purpurea* and *M. pudica*, whereas for the other plants, observations were made for six plants each.

### 2.3. Collection of Foam and Preservation

The foam produced by *P. costalis* nymphs was collected from various plants in Eppendorf tubes (1.5 mL capacity) and kept in a laminar air-flow chamber for 5–10 min, in order to sterilize the foam with UV-C germicidal lamp. The foam (usually 10 mL) was mixed with 10 mL of high-performance liquid chromatography (HPLC) water combined with acetone at a 1:1 ratio and then filtered through Whatman paper No. 1, before being stored at −20 °C until use.

### 2.4. Chemistry of Foam

#### 2.4.1. Qualitative Profiling of Carbohydrates

Four tests (Bial, Benedict, mucic acid, and Fehling) were performed to detect the presence of carbohydrates in the foam produced by *P. costalis*. Bial’s test was performed to detect the presence of pentoses: 5 mL of Bial’s reagent was added to 2–3 mL of solution, and the mixture was gently warmed under a low flame. The appearance of a blue color indicated the presence of pentoses. Benedict’s test was used to detect reducing sugars: five drops of concentrated hydrochloric acid were added to 5 mL of test solution and heated (50 °C) for 5 min in boiling water. Then, 10% sodium hydroxide solution was added to the mixture to produce a slightly alkaline solution (tested with red litmus paper). Benedict’s test was then carried out on this hydrolyzed solution. A red or yellow appearance indicated the presence of carbohydrates. The mucic acid test was performed to detect the presence of galactose: 0.2 mL of concentrated nitric acid was added to the test solution, which was then placed in a boiling water bath until the acid fumes were expelled. A few drops of water were then added. The formation of crystals indicated the presence of galactose. The Fehling test was performed to detect the presence of reducing sugars and non-reducing sugars: 1 mL of Fehling’s solution A (an aqueous solution of CuSO_4_) and 1 mL of Fehling solution B (a solution of potassium tartrate) were added to 2 mL of the test solution, mixed well and boiled. The formation of a reddish-brown precipitate indicated the presence of reducing sugars.

#### 2.4.2. Qualitative Profiling of Amino Acids

Six tests were performed for the detection of protein and amino acids (the Biuret reaction, the xanthoproteic reaction ninhydrin test, Hopkin’s Cole Test, the Sakaguchi reaction, and the sulfur test) [17] in the foam produced by *P. costalis*. The Biuret reaction was performed to detect the presence of a peptide bond: 2 mL of 10% sodium hydroxide and two drops of 0.1% copper sulfate solution were added to 2 mL of the test solution. A violet or pink color revealed the presence of proteins. The ninhydrin test was performed to detect the presence of amines: 1 mL of 0.1% ninhydrin solution was added to 4 mLof the solution at neutral pH and boiled for 2–3 min. A purple color indicated the presence of amines (e.g., proline). The xanthoproteic reaction was used to detect the presence of soluble protein:1 mL of nitric acid was added to 5mL of the test solution, which was then boiled. After cooling it, an excess of 40% sodium hydroxide was added to the mixture, and an orange color indicated the presence of phenylalanine. Hopkin’s Cole reaction was performed to detect the presence of tryptophan in proteins: 2 mL of glacial acetic acid and then 2 mL of sulfuric acid were added to 2 mL of the test solution. Color changes at the junction of the two liquids indicated the presence of tryptophan. The Sakaguchi reaction was performed to detect the presence of arginine in proteins: 1 mL of 10% sodium hydroxide solution and 1mL of 0.02% α-naphthol solution were added to 5 mL of the solution cooled on ice. After 3 min, 10 drops of alkaline hypobromide solution was added to the mixture, and the formation of a violet ring at the junction indicated the presence of arginine. Sulphur test was done to detect the presence of sulphur amino acids such as cysteine and cystine. For that, 2 mL of 40% sodium hydroxide and 10 drops of 2% lead acetate solution were added to 2 mL of the test solution. The mixture was boiled for a minute and then cooled. A black precipitate indicated the presence of sulfur-containing amino acids.

#### 2.4.3. Qualitative Enzyme Profiling

The presence or absence of trypsin, pepsin, protease, and polypeptidase [18], and invertase, lipase, and maltase [19] was determined using standard procedures.

#### 2.4.4. Gas Chromatography–Mass Spectroscopy (GC-MS)

GC-MS analysis was carried out on column chromatographic fractions. The analysis of the fractions and formulations was performed using the GC Clarus 500 Perkin Elmer system comprising an AOC-20i auto sampler and gas chromatographer interfaced to a mass spectrometer instrument equipped with an Elite-5MS fused capillary column (length: 300 m, diameter: 0.25 m, and film thickness: 0.25 μm). For GC-MS detection, an electron ionization energy of 70 eV was used. Helium (99.9%) was used as the carrier gas at a constant flow rate of 30 cm/second, and an injection volume of 3 μL was employed (split ratio: 10:1). The injector and ion-source temperature were 250 and 280 °C, respectively. The oven temperature was programmed to start at 110 °C (isothermal for 2 min), and then increase at 10 °C/min to 200 °C, before changing at 5 °C/minto 280 °C, ending with an isothermal condition at 280 °C for the acquisition of mass spectra and chromatograms with a Turbomass 5.2 (PerkinElmer, Inc., Shelton, CT, USA).

#### 2.4.5. Fourier Transform Infrared Spectroscopy (FT-IR)

Fourier transform infrared spectroscopy was used to identify the characteristic functional group present in the crystal. The solid crystal was analyzed by making transparent potassium bromide (KBr) pellets in order to prepare a translucent sample disc. Then, the disc was placed in a sample cup of diffuse reflectance. The infrared spectrum was obtained using an infrared spectrometer (FT-IR-8400S, Shimadzu, Columbia, MD, USA), and the sample was scanned in the range of 4000 to 400 cm, with a resolution of 4 cm.

#### 2.4.6. High-Performance Liquid Chromatography (HPLC)

Prior to HPLC analysis, an extracted dye solution was prepared using analytical grade solvents. The dyes were extracted from fibers of about 1 cm of dyed thread according to the procedure appropriate for each chemical class of dye. A red dye from a mordant dye from a plant or insect source was treated for a few minutes with equal portions of 3 mol hydrochloric acid and methanol at 100 °C. The dye solution was then evaporated to remove the acid and then resolved in about 0.25 mL of methanol for plant dyeing or purple molluscan dyeing. The yarn was treated for about 1 min with near-boiling NN-dimethyl formamide (DMF) to strip the vat dye. Each extracted dye solution was subsequently filtered through a 0.45 μm PTFE or nylon syringe filter. A 1μLfoam sample was mixed with 1 μL of HPLC water and injected, and the absorbance was read at 246 nm. A similar methodology was followed for the standard palmitic acid. The peaks were identified by their retention times (RTs) in comparison to the RT for palmitic acid.

### 2.5. Antimicrobial Activity

#### 2.5.1. Preparation of Potato Agar Medium (PDA)

Mycological agent agar (20 g) and 10 g of dextrose (HiMedia, Mumbai, India) were added to the filtrate, and the mixture was stirred until the dextrose was completely dissolved. The suspension was diluted with tap water, making it up to 1 L, and the final medium was autoclaved at 121 °C/15 LBS for 15 min. Cooled chloramphenicol (1g/L) was added to suppress bacterial growth.

#### 2.5.2. Microbial Culture

Various bacteria, such as *Xanthomonas axonopodis* pv. *malvacearum*, and *Xanthomonas citri* responsible for plant diseases; *Escherichia coli* and *Staphylococcus aureusis*, which are important causes of human infections; and the non-pathogenic bacterium *Pseudomonas fluorescens* were obtained from the Crop Protection Research Centre (CPRC) and maintained on nutrient agar slants with sub-culture for antibacterial activity. *Fusarium oxysporum*, the agent of a soil-borne plant disease, and *Candida albicans*, an agent of human disease, were obtained from the available stock of CPRC. The cultures were maintained on potato dextrose agar (PDA) slants and sub-cultured in Petri dishes prior to testing for antifungal activity.

#### 2.5.3. Agar Well Plate Method—Zone of Inhibition

The microbial activity was evaluated using the agar well diffusion technique [20]. For growth inhibition studies, sterile Petri plates (9 cm diameter and 1 cm height) were prepared with 20 mL each of sterile Mueller–Hinton agar (MHA) and potato dextrose agar (PDA). The medium was poured into Petri plates. After solidifying the media, 100 μL of various bacteria of *E. coli*, *S. aureus*, *X. malvacearum*, *X. citri* and *P. fluorescens* were used to inoculate separate MHA medium, and two different fungi, *C. albicans* and *F. oxysporum*, were used to inoculate separate PDA medium at normal room temperature using a sterile L-rod. After inoculating the media, 6 mm-sized wells were made using a sterile cork borer under aseptic conditions. A 100 μL test sample (12.5, 25, 50 and 100% of foam) and positive and negative controls were added. Standard antimicrobial agents and the standard antibiotic chloramphenicol (0.03%) were used as the antibacterial and antifungal agents, respectively. The negative control was acetone. Three replicates were performed for each concentration. The formation of clear zones of inhibition was recorded after 24 h for the bacteria and 72 h for the fungi. The cultures were maintained at 27 ± 2 °C in a BOD incubator (Kemi, Mudickal, India). The relative percentage inhibitions (PIs) for the test extract with respect to the positive control were calculated by using the following formula [21]:

$$\mathrm{PI}=\frac{100\times \left(x-y\right)}{\left(z-y\right)}$$

where

*x* = the total area of inhibition for the test extract;
*y* = the area of inhibition for the negative control;
*z* = the area of inhibition for the positive control.

### 2.6. Statistical Analysis

The data for the foam positions for various plants, foam size on various plants, and temperature differences between the outside and inside of the foam were subjected to ANOVA, and their significance is expressed at the 5% level. The normality and homogeneity of the variances of the data were first tested, and data not meeting these criteria were log- or arcsin-transformed. The responses of different concentrations of spittlebug foam (100, 50, 25 and 12.5%) to *E. coli*, *X. malvacearum*, *X. citri*, *S. aureus*, *P. fluorescens*, *C. albicans*, *F. oxysporum*, chloramphenicol and acetone were also subjected to ANOVA, and their significance is expressed at the 5% level. The biofoam length and breadth, production height and surface area were correlated and the R^2^ was calculated using the same software. The software IBM SPSS Statistics version 25 (IBM Corp, Armonk, NY, USA) was used for the data analysis.

## 3. Results

### 3.1. Distribution of Foam Onvarious Plants and Their Parts

**Figure 1:**
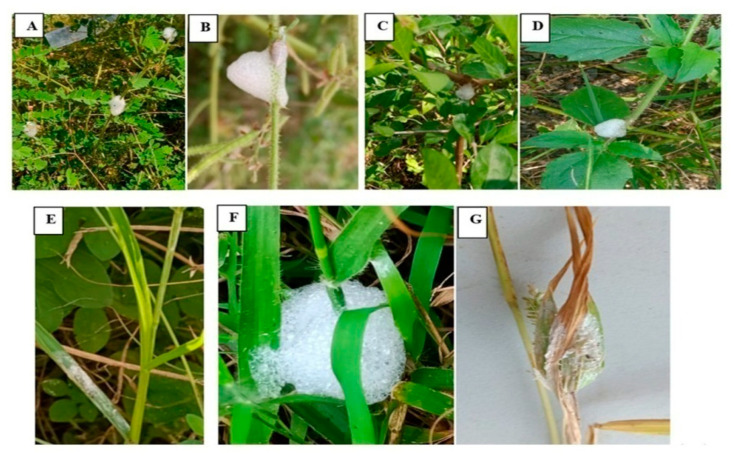
Production of foam by nymphal stages of *P. costalis* on *T. purpurea* (**A**,**B**), *M. pudica* (**C**), *T. procumbens* (**D**), and grasses (**E**–**G**). Discoloration of grass leaf blades (**E**) and joining of both leaf blades and inflorescence (**F**).

The foam produced by *P. costalis* nymphs on11 plant species was recorded. The nymphs of this spittlebug produced their foams at the stems (Figure 1A–D), leaves, and nodes of *B. cylindrica*, *C. dactylon*, *C. olitorius*, *L. inermis*, *P. amarus*, *C. ternatea*, *T. purpurea* (Figure 1A,B), *M. pudica* (Figure 1C), *T. procumbens* (Figure 1D), *O. americanum* and *E. amblilies* (Table 1). In grasses, *P. costalis* nymphs produced foam on leaf blades (Figure 1E), nodes (Figure 1E) or the inflorescence (Figure 1F). The foam affects plant growth by reducing photosynthetic areas (Figure 1F,G). However, *P. costalis*’ foam was most commonly observed on Fabaceae plants such as *T. purpurea* and *M. pudica* (15.42% of each).

Most of the foams included the fourth instar nymphs (92.5%) of *P. costalis*. Some of the temperature data were taken for foams containing fifth instar nymphs (7.5%) of *P. costalis*. The *P. costalis* spittle was significantly (*p* < 0.000) more abundant at the stems (64%) than the nodes (30%), and leaf and internode regions (6%) of the plants observed. The plant preference of spittlebugs depends on different factors, of which feed is one (Table 1).

Even the height at which the spittlebug produced the foam varied from plant to plant. Similarly, the length (1.00 ± 0.11 to 3.88 ± 0.77 cm), breadth (0.61 ± 0.10 to 4.68 ± 0.34 cm) and size (0.61 to 7.84 cm^2^) of the spittle also varied (Table 2). In *L. inermis* a tall shrub plants, *P. costalis* made much foam in the nodes and stems, because of its glabrous and multi branched nature and also a type of chemical interaction between the plant and insect. A significantly positive correlation (R^2^ = 0.912) was observed between the length and breadth of the foam produced by *P. costalis* on various plant species. However, the height of the site at which the foam was produced, and the surface area showed more correlation (R^2^ = 0.0005). This shows that *P. costalis* nymphs feed systematically without discriminating between plants and positions.

### 3.2. Ecophysiology of Foam and Spittlebug

In most of the plants, the temperatures outside the foam produced by *P. costalis* and in the external environment were significantly different (df = 3,212; F = 374.58; *p* < 0.0001) (Table 3). The *results revealed* a significant temperature variation between the outside and inside of the foam produced by nymphs of *P. costalis* (df = 53; F = 5.3972; *p* < 0.0001). However, this was not the case for Fabaceae such as T. purpurea (df = 1,30; F = 0.7009; *p* > 0.05) and *M. pudica* (df = 1,30; F = 0.6990; *p* > 0.05).

### 3.3. Chemical Composition of Foam

The carbohydrates, amino acids, proteins and enzymes were qualitatively profiled, and the results are presented in Table 4, from which it is very clear that carbohydrates, proteins, amino acids (phenylalanine) and enzymes (lipase, trypsin, maltose and protease) are present in the spittlebug foam.

**Figure 2:**
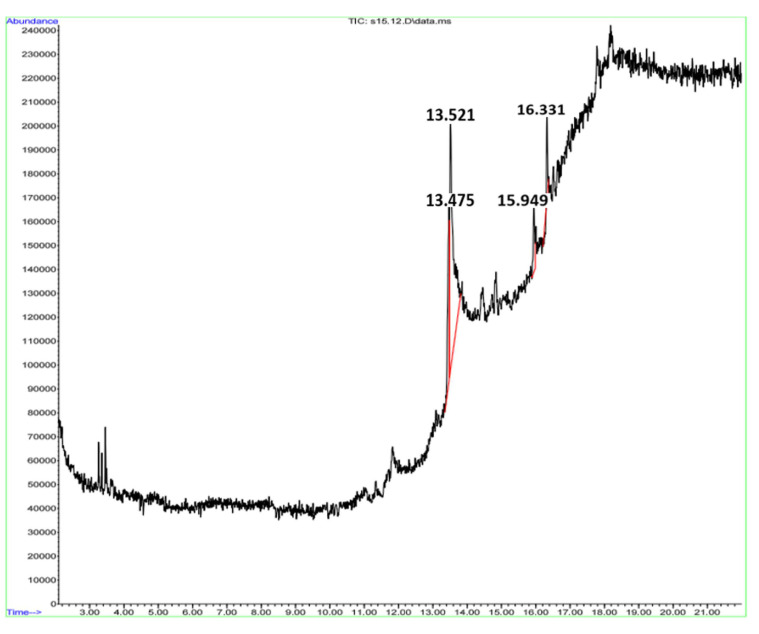
Gas chromatography–mass spectrum of *P. costalis* foam.

GC-MS analyses showed the presence of long chain fatty acids (88.33%) along with other minor compounds (Table 5 and Figure 2). The highest proportions were found for long-chain fatty acids such as 6-octadecanoic acid,(E)-(68.43%) and octadec-9-enoic acid (19.90%), a biomolecule practically insoluble (in water) and acidic.

**Figure 3:**
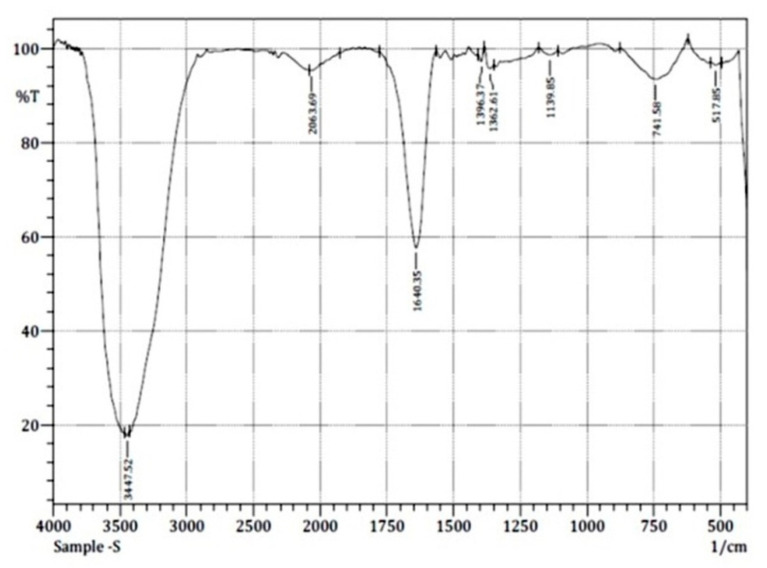
Fourier transform infrared spectroscopy spectrum of *P. costalis* foam.

The above-mentioned compounds’ functional groups were confirmed by FT-IR analyses (Table 6 and Figure 3) with the vibrations from 517.85 cm^−1^ to 3447.52 cm^−1^. 9-Octodecanoic acid,(E)- and 6-Octodecanoic acid,(E)-(C_18_H_34_O_2_) (common name, stearic acid) (C_18_) constitute the major compounds of the foam (88.33%) (Table 6).

**Figure 4:**
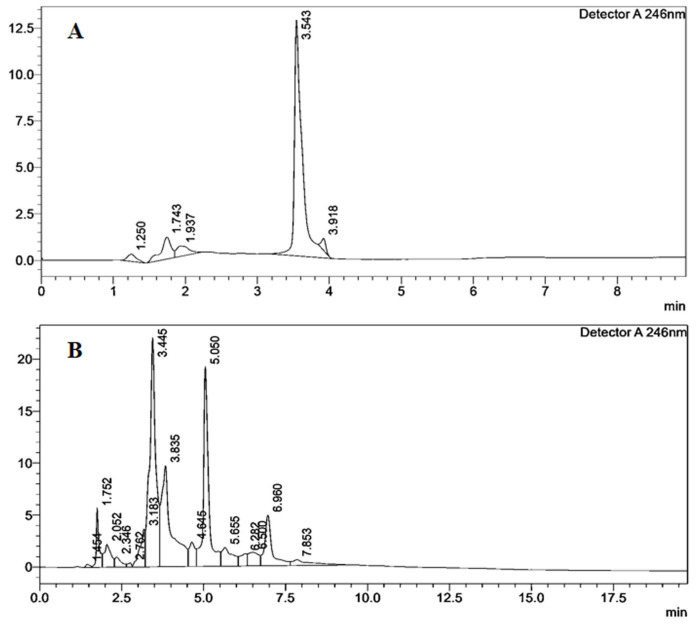
High-performance liquid chromatography (HPLC) chromatogram of palmitic acid (A) and spittlebug biofoam *P. costalis* (**B**) at 246 nm.

The HPLC spectra reveal the presence of many fatty acids in spittlebug foam with RTs ranging from 1.454 to 7.853 min (Table 7; Figure 4).

### 3.4. Antimicrobial Activity of the Foam

#### 3.4.1. Human Pathogens

**Figure 5:**
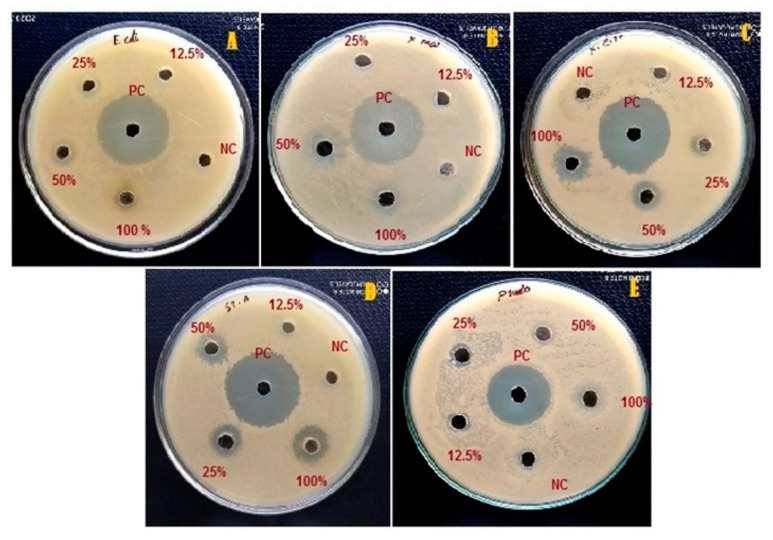
Impact of P. costalis foam concentrations (12.5, 25, 50 and 100%) on the growth of *Escherichia coli* (**A**), *Xanthomonas malvacearum* (**B**), *Xanthomonas citri* (**C**), *Staphylococcus aureus* (**D**) and *Pseudomonas fluorescens* (**E**).

In the present investigation, the antibacterial activity of the foam produced by *P. costalis* nymphs was observed (Table 8 and Table 9; Figure 5). Pure foam (100%) caused a significant (df = 3,8; F = 14.17; *p* < 0.001) reduction in *E. coli*, with 43.26 ± 1.18% growth inhibition, and its effect gradually declined as the foam concentration decreased. *S. aureus* isa major bacterial human pathogen that causes a wide variety of clinical manifestations. This study showed that a higher concretion (100%) of foam reduced the growth of *S. aureus* by 55.89 ± 3.85%. However, more studies are needed before this material can be utilized for *S. aureus* management. All the tested concentrations of *P. costalis* foam did not affect the growth of *Fusarium oxysporum* and *Candida albicans*. Furthermore, comparisons between 100% and 50% (*p* < 0.01), 100% and 25% (*p* < 0.01), 100% and 12.5% (*p* < 0.01), 50% and 12.5% (*p* < 0.01) and 25% and 12.5% (*p* < 0.05) foam showed significant differences (*p* < 0.05).

#### 3.4.2. Plant Phytopathogens

Spittlebug foam dose-dependently reduced the growth of *Xanthomonas axonopodis* pv. *malvacearum* at concentrations ranging from 100% (46.74 ± 2.09%) to 12.5% (10.65 ± 3.43%) (Table 9).

#### 3.4.3. Biosafety to a Bacterium

Spittlebug foam liquid suppressed *P. fluorescens* growth in a dose-dependent manner (Table 8 and Table 9). For instance, the zone of inhibition for 100% foam was 16.0 ± 1.0 cm, and that for 12.5% was 5.33 ± 0.7 cm (Table 9). The ANOVA results show a significant reduction in *P. fluorescens* by spittlebug foam (df = 3,8; F = 79.7; *p* < 0.0001).

## 4. Discussion

It is known that *P. costalis* nymphal stages are highly polyphagous feeds on 37 host plants of 10 families, but they prefer plants of the Asteraceae [1]. Spittlebugs prefer grass, herbaceous species, bushes and trees [4] for the production of foams, as observed in this study. Furthermore, it has been reported that the host-plant preference of nymphs varies according to the region and nymphal instar for *Philaenus spumarius *L., *Neophilaenus campestris* (Fallén), and *Aphrophora alni* (L.) [16], as well as varying over time. Volatile compounds from grasses also influence the feeding of *Mahanarva spectabilis *(Distant) [22].

The difference between the interior foam temperature and external temperature might be due to the favorable humidity prevalent around this family of plants. A similar observation was also recorded for *Mahanarva fimbriolata*, and it was reported that the foam is useful for thermoregulation or defense in the spittlebugs [13]. Future investigations on the physical properties of the foam, especially optical reflection and heat dissipation, will provide further insights.

Lipids [23], proteins [24] and carbohydrates [25] play important roles in the stabilization of the foam and also in maintaining a stable bubble layer around the nymphs. In addition, the foam consists of water obtained from the plant sap [26,27]. The proportions of these compounds should be determined in a future study.

The foam of the spittlebug is considered an adhesive secretion [27]. Two minor compounds were recorded, benzo(h)quinoline, 2–4 dimethyle-(7.70%) and 2,4,6-cycloheptatrien-1-one,3,5-bis-trimethylsilyl (3.97%). Eisner and co-workers [28] reported benzo(h)quinoline as a defense molecule secreted by arthropods. GC-MS analyses of the mucus of three common land snails, *Eobania vermiculata*, *Theba pisana* and *Monacha obstructa*, showed the presence of 1 cyclotrisiloxane, hexamethyl [29]. 2,4,6-Cycloheptatrien-1-one, 3,5-bis-trimethylsilyl (3.97%), considered a microbicidal compound, has been recorded in many plants such as *Psidium guajava* [30], and it is also considered a nematocidal compound [31].

The chemistry of spittlebug biofoam is poorly understood [32] but comprises a complex mixture of polypeptides, proteoglycans and calcium; glycoproteins [32]; proteins, carbohydrates and lipids [23,24,25,26,33]; palmitic acid and stearic acid [13] and fatty acid-derived alcohols, γ-lactones and 1-monoacylglycerol, as well as polyolpinitol and the polyhydroxy alkanoate poly-3-hydroxybutyrate [11]. The mineral composition of the foam resembles that of the xylem sap upon which the insect feeds [32,34]. This study shows the presence of carbohydrates, proteins, amino acids, enzymes, and octodecanoic acid and confirms the literature cited here. In addition to the C_18_ fatty acid, another essential fatty acid, palmitic acid (C_16_), has been reported in spittlebug foam [13]. Palmitic acid and stearic acid are the major products of fatty acid synthase. Both are saturated; that is, they contain no double bonds between adjacent carbons. Many fatty acids are also recorded in the defensive secretions of insects. The more common fatty acids in defensive secretions are C_12_ to C_18_ saturated and unsaturated compounds [35], as observed here. The analysis of native and underivatized lipids within body fluids is still a challenging task, particularly if the molecular structures of individual components need to be identified quickly. The lipids consist of different major classes such as fatty acids, neutral lipids, and lipids with positively or negatively charged head groups, with manifold structurally diverse subclasses. Variations in lipid composition have been attributed to different pathologies such as neoplastic and neurodegenerative diseases and diabetes mellitus, among many. Furthermore, some lipid classes are involved in cell death (apoptosis and necrosis) and cellular signaling and are precursors for lysophospholipids, diacylglycerols, and phosphatic and arachidonic acid. Gas chromatography (GC) [36] and high-performance liquid chromatography (HPLC) [37] are commonly used for lipid analysis.

**Figure 6:**
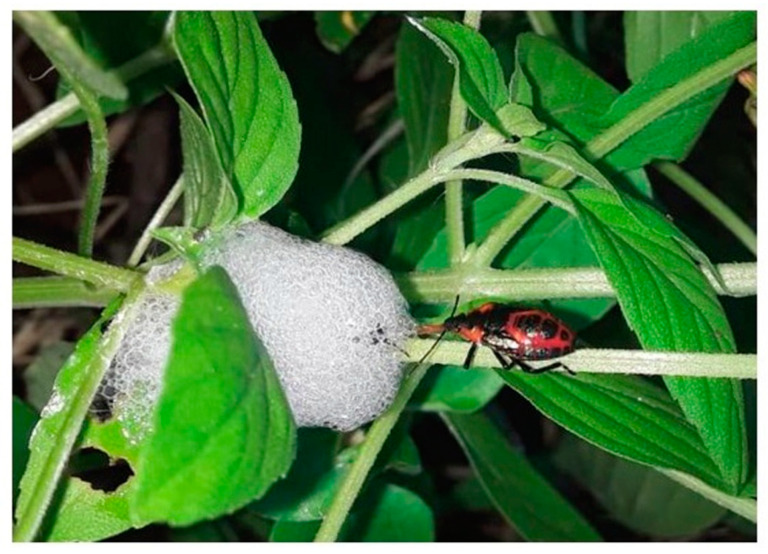
Feeding of a Pyrrhocoridae predator, *Antilochus coqueberti* (Heteroptera) nymph, on *P. costalis* nymph under field conditions.

Beneficial arthropods, such as carabids, staphylinids, mites, spiders, tiger beetles, and ants in turf grass habitats have been reported in the literature [38,39]. During our frequent field visits, we observed, for the first time to the best of our knowledge, a Pyrrhocoridae predator *Antilochus coqueberti* (Heteroptera) nymphs—feeding on *P. costalis* nymphs, present inside the spittle (Figure 6). Though the foam is produced to protect the spittlebugs from their natural enemies [9], *A. coqueberti* is thought to predate *P. costalis* nymphs. Elaborate studies are needed to determine the type of interaction between this pest and this predator.

Most insects have self-defense systems protecting against invading microorganisms, as recorded in vertebrates. Spittlebug foam also contains many microbes that protect them from surrounding environments [12]. Unique silkworm excretory red fluorescent proteins (SE-RFPs) bearing tetrapyrrole moieties have been reported [40,41]. The authors of [42,43] also showed antibacterial activity in insect and earworm surface excreta, respectively. *Xanthomonas citri* is a bacterial pathogen that causes citrus canker—a disease that results in heavy economic losses to the citrus industry worldwide, in terms of damage to trees, reduced access to export markets, or the costs of its prevention and control. Interactions between *X. citri* and antagonistic bacteria including *Bacillus subtilis*, *B. polymyxa*, *Pseudomonas fluorescens* and *Serratia marcescens* [44], *Erwiniaher bicola* [45], *Pseudomonas* sp. [46] and *P. fluorescens* [47] have been reported in vitroandin vivo. However, the practical usefulness of these bacteria in controlling the pathogen has not been proved. Our results prove that spittlebug foam can be utilized for the management of *X. citri* (Table 8 and Table 9).

Previous studies have shown that the foam of *P. costalis* can repress the growth of *Fusarium oxysporum* [14] due to the presence of three chitinases: β-*N*-acetylglucosaminidase, chitobiosidase and endochitinase. However, our study revealedthat the foams produced by the nymphs of *P. costalis* at various tested concentrations did not affect the growth of *F. oxysporum* or the human pathogen *C. albicans*. In another study, the antagonistic bacterium *Stenotrophomonas maltophilia* and *Delftia tsuruhatensis* isolated from the froth of the spittlebug *P. costalis* showed antifungal activity against the phytopathogenic fungi *F. oxysporum* f.sp. *pisi* and *Colletotrichum gloeosporioides* Penz. [48]. *Xanthomonas axonopodis* pv. *malvacearum* causes bacterial blightin cotton plants [49] and is currently managed with chemicals [50]. However, spittlebug foam is considered a novel material for managing this pathogen.

*Pseudomonas fluorescens* has been utilized as an antagonistic bacterium against many phytopathogenic bacterium such as *X. citri* [47] and various *Pseudomonas* species have been shown to induce plant growth [51]. A number of studies have explored the substantial biological activities of meadow spittlebug *Philaenus spumarius *(L.) (Cercopide: Homoptera) on *Alhagi pseudalhagi *(M. Bieb.) Desv. [52]. This study also proves the antimicrobial activity of the spittlebug for both pathogenic and non-pathogenic microbes.

## 5. Conclusions

Our study showed that *P. costalis* produced its foam on either monocotyledons or dicot plants in its vicinity and was most commonly observed in *T. purpurea* and *M. budica*. The nymphs preferred to feed on stems, followed by nodes and leaves. The foam size varied in relation to the type of plant part selected for production, being larger for *L. inermis* than the other plants. The temperature inside the foam is lower than the external temperature, which is helpful for development. Chemically speaking, the foam consisted of carbohydrates, amino acids (trypsin), enzymes (protease), and fatty acids (octodecanoicacid) and also contained water collected from the plant sap. The foam showed antibacterial activity against *E. coli*, *X. malvacearum*, *X. citri*, *S. aureus* and *P. fluorescens*.

## Figures and Tables

**Table 1:** Locations of *P. costalis* foam on different plants. N indicates number of spittle specimens observed.

Plants	N	Foam Position (%)			No. of Nymphs
		Node	Stem	Leaves	
*Boehmeria cylindrical* L.	6	100	-	-	1
*Cynodon dactylon* L.	20	30	10	60	2
*Corchorus olitorius* L.	26	53.84	38.46	7.69	3
*Lawsonia inermis* L.	9	55.55	44.44	-	3
*Tridax procumbens* L.	17	-	100	-	1
*Phyllanthus amarus* L.	14	-	100	-	1
*Clitoria ternatea* L.	9	-	100	-	1
*Tephrosia purpurea* L.	31	-	100	-	1
*Mimosa pudica* L.	31	-	100	-	1
*Ocimum americanum* L.	25	60	40	-	3
*Eragrostis amabilis* L.	13	30.76	69.23	-	3
Mean		30.01 ± 1.02	63.83 ± 1.17	6.15 ± 0.54	1.81
ANOVA		df = 10; F = 9,27; *p* = 0.000			
		Node–Stem	Node–Leaf	Stem–Leaf	
		*p* < 0.05	*p >* 0.05	*p* < 0.01	

**Table 2:** Length and breadth (cm) of foam produced by *P. costalis* on various plants available on St. Xavier’s College campus.

Plant Name	Foam Produced Height (cm)	Number of Foam(s)/Plant	Foam Size (cm)		Surface Area (cm^2^)
			Length	Breadth	
*Boehmeria cylindrica*	45.17 ± 6.31	1.6 ± 0.3	1.12 ± 0.72	0.98 ± 0.16	1.11
*Cynodon dactylon*	7.65 ± 0.97	1.2 ± 0.1	1.00 ± 0.11	0.61 ± 0.10	0.61
*Corchorus olitorius*	15.78 ± 5.22	1.8 ± 0.3	1.32 ± 0.11	1.19 ± 0.17	1.57
*Lawsonia inermis*	19.67 ± 1.19	1.5 ± 0.2	3.88 ± 0.77	4.68 ± 0.34	18.15
*Tridax procumbens*	7.23 ± 0.83	1.8 ± 0.2	1.19 ± 0.57	0.91 ± 0.21	1.08
*Phyllanthus amarus*	5.71 ± 0.61	1.5 ± 0.1	1.01 ± 0.77	0.74 ± 0.11	0.74
*Clitoria ternatea*	29.20 ± 1.95	1.9 ± 0.3	1.26 ± 0.11	0.93 ± 0.01	1.23
*Tephrosia purpurea*	33.10 ± 2.76	3.2 ± 0.2	1.30 ± 0.51	1.01 ± 0.1	1.31
*Mimosa budica*	20.89 ± 3.49	3.0 ± 0.3	1.22 ± 0.32	0.92 ± 0.15	1.12
*Ocimum americanum*	21.20 ± 0.62	1.9 ± 0.2	1.82 ± 0.51	1.68 ± 0.86	3.06
*Eragrostis amabilis*	23.92 ± 0.71	2.1 ± 0.1	3.24 ± 0.18	2.42 ± 0.89	7.84
			df = 2,30; F = 1.28; *p* = 0.292788		

**Table 3:** Foam inner temperature (°C) in relation to environmental humidity (%) and temperatures (°C).

Humidity (%)	Temperature (°C)		Temperature Difference
	Environment	Inner Side of the Foam	
**Other plants (cumulation of nine plant species)**			
72.42 ± 0.14	31.42 ± 0.18	26.37 ± 0.17	4.87 ± 0.34
F = 16.5075	7.472	2.0985	5.3972
df = 53	53	53	53
*p* < 0.0001	<0.0001	<0.05	<0.0001
ANOVA: df = 3,212; F = 374.58; *p* < 0.0001			
*Tephrosia purpurea*			
69.91 ± 0.07	35.23 ± 0.05	34.30 ± 0.06	0.96 ± 0.05
df = 30	30	30	30
F = −1.358	95.6362	127.95	0.7009
*p* > 0.05	<0.0001	<0.0001	˃0.05
ANOVA: df = 3,120; F = 127.95; *p* < 0.0001			
*Mimosa pudica*			
71.32 ± 0.23	35.13 ± 0.04	34.22 ± 0.05	0.95 ± 0.06
df = 30	30	30	30
F = −1.300	92.0039	121.0015	0.6990
*p* > 0.05	<0.0001	<0.0001	>0.05
ANOVA: df = 3,120; F = 127.95; *p* < 0.0001			

**Table 4:** Qualitative chemical screening of the foam produced by *P. costalis* nymphs.

Biomolecules	Tests	Results
Carbohydrates		
	Bial	++
	Benedict	+
	Mucic acid	+
	Fehling	+++
Proteins		
	Biuret	+++
	Ninhydrin	-
	Xanthoproteic	+
	Sakaguchi	-
	Sulfur	-
	Hopkin’s Cole	-
Enzymes		
	Invertase	-
	Maltose	++
	Polypeptidase	-
	Protease	++
	Lipase	-
	Hyaluronidase	-
	Trypsin	+
	Pepsin	-

- indicates absent and + indicates present; +++—highly intense, ++—moderately intense and + slightly positive.

**Table 5:** GC-MS analysis of spittlebug *P. costalis* foam.

Peak No.	Retention Time (min)	Area	Area (%)	Proposed Compound	Library Reference Number
1	13.471	2,102,798	19.90	9-Octadecanoic acid,(E)-	129353
2	13.518	7,230,682	68.43	6-Octadecanoic acid,(E)-	129340
3	15.948	813,865	7.70	Benzo(h)quinoline, 2-4 dimethyle-	67018
4	16.327	419,267	3.97	2,4,6-Cycloheptatrien-1-one, 3,5-bis-trimethylsilyl	102104

**Table 6:** FT-IR analyses of spittlebug *P. costalis* foam.

Peak (cm^−1^)	Appearance	Intensity	Corrected Area	Group	Compound Class
517.85	Weak	0.617	0.052	C-Br stretch	Alkyl halides
741.58	Weak, broad	7.704	5.064	N-H stretch	Threonine
1139.85	Weak	1.1448	0.22	C-O stretch	Carbonate groups
1362.61	Weak	2.311	0.259	-C=O	Carbonate groups
1415.65	Weak	2.396	0.129	C-C stretch	Aromatics
1640.35	Medium Sharp	41.824	18.675	C=C stretch	Conjugated alkene
2063.69	Weak	0.49	0.349	S-H stretch	Thiol
3447.52	Strong, sharp	0.912	0.436	O-H stretch	Alcohol

**Table 7:** High-performance liquid chromatography (HPLC) analysis of palmitic acid and spittlebug *P. costalis* foam at 246 nm.

Peak No.	Retention Time (min)	Area	Height
**Palmitic acid**			
1	1.250	3440	390
2	1.743	11,769	1185
3	1.937	6686	551
4	3.543	95,073	12,690
5	3.918	2527	641
**Spittlebug foam**			
1	1.454	2623	292
2	1.752	32,838	5594
3	2.052	32,146	2188
4	2.346	13,185	983
5	2.762	3370	399
6	3.183	40,122	3649
7	3.445	285,463	21,987
8	3.835	207,627	9688
9	4.645	29,666	2347
10	5.050	211,510	19,126
11	5.655	41,340	1798
12	6.282	18,098	1180
13	6.500	28,804	1307
14	6.960	76,212	4874
15	7.853	22,984	561

**Table 8:** Impact of different concentrations of *P. costalis* foam (100, 50, 25 and 12.5%) on antibacterial activity against *Escherichia coli*, *Xanthomonas malvacearum*, *Xanthomonas citri*, *Staphylococcus aureus* and *Pseudomonas fluorescens*. Chloramphenicol was the positive control, whereas acetone was the negative control. Same lowercase alphabet for each microbes was statistically significant at 5%.

Microbes	Spittlebug Foam (%)	Zone of Inhibition (cm)
*Escherichia coli*	100	14.3 ± 0.3 b
	50	11.7 ± 0.7 c
	25	10.0 ± 0.6 c
	12.5	8.0 ± 0.5 d
	Chloramphenicol	16.1 ± 0.8 a
	Acetone	4.0 ± 0.2 e
*Staphylococcus aureus*	100	17.0 ± 1.0 b
	50	11.7 ± 0.3 c
	25	10.0 ± 1.0 c
	12.5	6.6 ± 0.3 d
	Chloramphenicol	25.6 ± 1.2 a
	Acetone	4.0 ± 0.2 e
*Xanthomonas malvacearum*	100	10.2 ± 0.5 b
	50	8.3 ± 0.4 c
	25	7.7 ± 0.8 c
	12.5	5.4 ± 0.5 d
	Chloramphenicol	15.7 ± 0.8 a
	Acetone	4.0 ± 0.2 e
*Xanthomonas citri*	100	15.7 ± 0.3 b
	50	11.7 ± 0.9 c
	25	10.6 ± 0.9 c
	12.5	7.6 ± 0.3 d
	Chloramphenicol	24.6 ± 1.2 a
	Acetone	4.0 ± 0.2 e
*Pseudomonas fluorescens*	100	16.0 ± 1.0 a
	50	12.7 ± 0.3 b
	25	7.3 ± 0.6 c
	12.5	5.3 ± 0.7 d
	Chloramphenicol	16.7 ± 0.3 a
	Acetone	4.0 ± 0.2 e

**Table 9:** Impact of *P. costalis* foam concentrations (100, 50, 25 and 12.5%) on the growth of chosen pathogenic microbes.

Foam Concentration (%)	Statistics	*E. coli*	*X. malvacearum*	*X. citri*	*S. aureus*	*P. fluorescens*
100	$\bar{x}$ ± SE	43.3 ± 1.2	46.7 ± 2.1	44.9 ± 2.9	55.9 ± 3.8	51.6 ± 2.2
	t	2.7627	3.2273	0.5078	4.1224	5.16
	*p*	0.0549	0.0510	0.3310	0.0270	0.0177
50	$\bar{x}$ ± SE	29.8 ± 4.8	36.6 ± 4.6	30.6 ± 6.9	28.9 ± 2.2	34.5 ± 1.5
	t	−0.044	1.4439	0.09	−0.4687	2.8947
	*p*	0.4844	0.1427	0.4682	0.3427	0.0507
25	$\bar{x}$ ± SE	21.2 ± 4.1	24.5 ± 4.4	25.4 ± 6.5	21.1 ± 5.9	24.2 ± 2.1
	t	−0.9222	−0.1179	0.0561	−0.6574	−0.3772
	*p*	0.2268	0.4584	0.4801	0.2892	0.3711
12.5	$\bar{x}$ ± SE	10.7 ± 3.5	10.6 ± 3.4	8.6 ± 1.8	3.3 ± 1.7	12.0 ± 1.5
	t	−4.0623	−4.1776	−3.5328	−0.373	5.3662
	*p*	0.0277	0.0264	0.0358	0.3724	0.0165
ANOVA	df	3,8	3,8	3,8	3,8	3,8
	F	14.17	17.01	4.72	32.6	79.7
	*p*	0.0014	0.0007	0.0352	0.0001	0.0001

## Data Availability

The data presented in this study are available on request from the corresponding author.
